# High gamma activity of 60–70 Hz in the area surrounding a cortical tuber in an infant with tuberous sclerosis

**DOI:** 10.1186/1824-7288-38-15

**Published:** 2012-05-03

**Authors:** Kaori Irahara, Eiji Nakagawa, Ryoko Honda, Kenji Sugai, Masayuki Sasaki, Takanobu Kaido, Yu Kaneko, Akio Takahashi, Taisuke Otsuki

**Affiliations:** 1 Department of Child Neurology, National Center Hospital, National Center of Neurology and Psychiatry (NCNP), 4-1-1 Ogawahigashi-cho, Kodaira, Tokyo, 187-8551, Japan; 2 Department of Pediatrics, Shimada Ryoiku Center, Tokyo, Japan; 3 Department of Neurosurgery, National Center Hospital, NCNP, Tokyo, Japan

**Keywords:** Tuberous sclerosis, Periodic spasms, Frequency oscillations, Multiple band frequency analysis, Scalp electroencephalogram

## Abstract

**Purpose:**

To detect the epileptogenic region causing epileptic spasms in an infant with tuberous sclerosis (TS).

**Methods:**

We applied a multiple band frequency analysis to video electroencephalographic (EEG) recordings of the infant’s scalp. We also performed computed tomography (CT), magnetic resonance imaging (MRI), single-photon emission computed tomography (SPECT), and magnetoencephalography (MEG) of the brain in order to ascertain the epileptic focus.

**Results:**

During the periodic spasms, we identified fast ictal activity with frequencies of 60–70 Hz in the right centroparietal region. This region was part of the area surrounding the largest cortical tuber that was identified on CT and MRI and was located in the right frontal lobe. An area of increased blood perfusion that was observed with SPECT and dipole sources that were determined with interictal MEG were also located in this area. In addition, ictal frequency oscillations (FOs) with high gamma activity were identified over the cortex surrounding the largest tuber. After a lesionectomy of this tuber, the periodic spasms disappeared, and no FOs were detected over this area.

**Conclusions:**

Scalp EEG, which identified the ictal onset zone by detecting fast activity that was suggestive of FOs, was useful for detecting the epileptogenic region in an infant with TS.

## Background

Tuberous sclerosis (TS) is a neurocutaneous syndrome involving multiple organs. Because of abnormalities in migration, proliferation, and differentiation, characteristic hamartomas are found in the skin, retina, kidney, lung, heart, and brain. One of the most important complications of TS is epilepsy, which occurs in 80%–90% of cases [1]. A common type of seizure is infantile spasms, which occur in approximately 30%–60% of patients with TS in the first month of life. Infantile spasms with localization-related epilepsies were first described as periodic spasms by Gobbi et al. [2]; they inferred that cortical mechanisms played a critical role in their pathophysiology. Recently, advances in digital electroencephalography (EEG) recording techniques have allowed recordings with high sampling frequencies. High-frequency oscillations (HFOs) with frequencies above 80 Hz that are observed on EEG are biomarkers of epileptogenesis, and they can be detected in focal cortical areas, implying that these are ictal onset zones [3,4]. Furthermore, high gamma activity with frequencies of 50–80 Hz also appear in epileptogenic zones with ictal discharge [5]. Therefore, not only HFOs, but also high gamma activity, may be useful for detecting epileptogenic zones. The detection of epileptogenic HFOs or high gamma activity, however, is typically performed using intracranial electrodes. Kobayashi et al. reported HFOs in scalp EEGs that were performed on patients with infantile spasms, and Yamazaki et al. observed fast activity in young children with hemimegalencephaly [6,7]. Fast activity was detected just before the onset of spasms, and these findings suggested that the cortex played a major role in generating epileptic spasms [4,5,8]. Moreover, ictal high gamma activity occurred with slow waves that had spasms that were less than 1 Hz, although the origin of the slow wave was unknown [9]. We describe the case of a 1-year-old girl with periodic spasms that were secondary to TS due to a *TSC1* gene mutation. We performed a scalp EEG with a high-sampling frequency and analyzed the ictal fast activity on the scalp EEG by multiple band frequency analysis (MBFA).

## Case report

**Figure 1:**
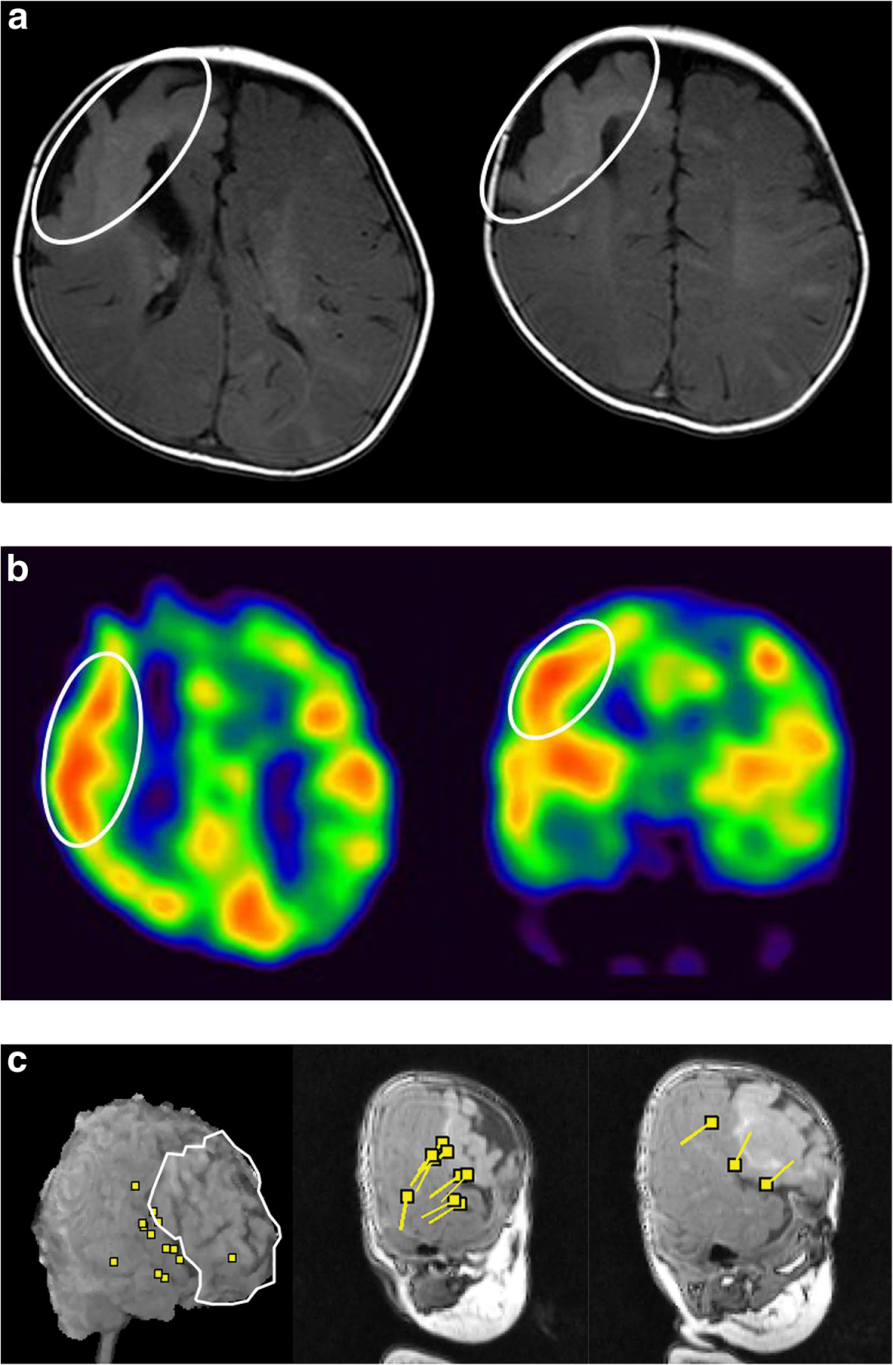
**Magnetic resonance imaging, single-photon emission computed tomography, and magnetoencephalography at the age of 4 months. (a)** Brain magnetic resonance imaging scan showing a slightly hyperintense lesion on T1-weighted images and cortical atrophy in the right frontal lobe involving the superior and medial frontal gyri and in the left parietal lobe. **(b)** Axial and coronal sections of ictal single-photon emission computed tomography scans. Increased perfusion was seen over the right central region (white encircled area). **(c)** A three-dimensional image and sagittal sections of the interictal magnetoencephalography scans. The region encircled by the white line indicates the cortical tuber. Spike dipoles were clustered at the pericortical tuber, and only one spike dipole was detected above the cortical tuber.

**Figure 2:**
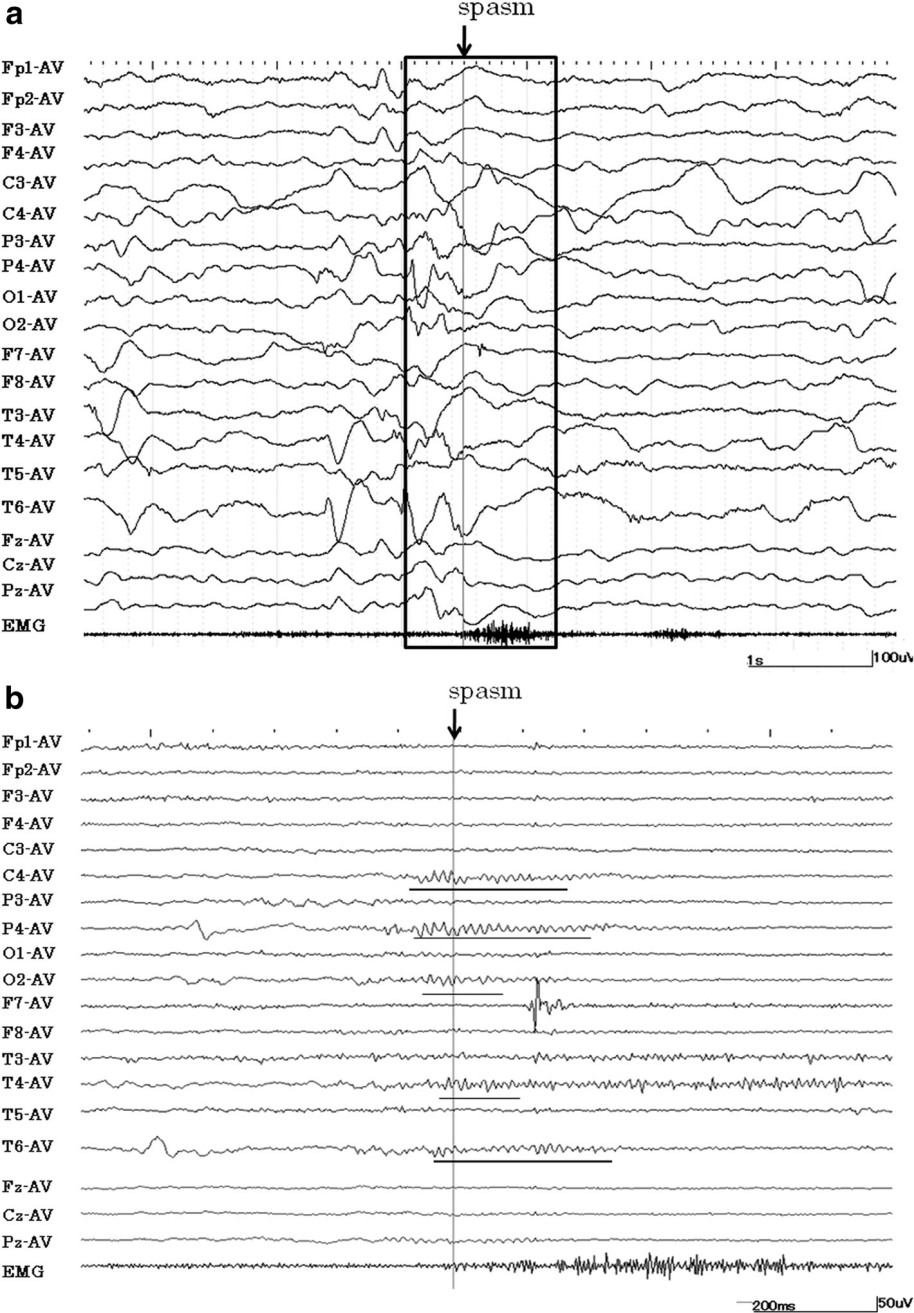
**Visible fast oscillations recorded around periodic spasms.** (**a**) Ictal encephalographic (EEG) recordings around the occurrence of periodic spasms. A mixture of sharp waves and slow waves were seen just before spasms at P4, T4, and T6 (time constant, 0.1 s). (**b**) Expansion of the EEG recording in Figure 2a encircled by the black line. Fast rhythmic waves were seen at C4, P4, T4, T6, and O2 (underlined) (band pass filter ranging from 5.7 to 120 Hz).

A 1-year-old girl was born uneventfully and at term. At 1 month of age, she experienced brief tonic seizures of the upper limbs with eye deviation several times a day. At 2 months of age, she developed spasms in clusters following a series of three or four brief tonic seizures a day. The patient was admitted to our hospital at the age of 4 months. At this point, zonisamide and clonazepam treatments were initiated. However, the spasms that occurred in clusters were refractory to these treatments and increased to approximately 10 times a day. Typically, each spasm episode was accompanied by the following: slightly tonic limb contraction, head adverse motion, and eye deviation to the right. These seizures lasted around 1 min. The periodic spasms that occurred in clusters consisted of a brief symmetrical contraction of the axial muscles that was associated with intense head nodding. At this point, she could gaze and track subjects. However, after the onset of this type of seizure, her development arrested. Computed tomography (CT) and magnetic resonance imaging (MRI) of the brain revealed a calcified lesion in the right frontal lobe and similar small calcified lesions in the left frontal lobe and subventricular regions. This massive tuber seemed to overlap the rolandic area (Figure 1a). DNA that was isolated from the patient’s blood revealed a heterozygous mutation, IVS29 + 1 G > A, which has been reported as a splicing mutation of intron 29. On the basis of these radiological findings and the genetic analysis, the patient was diagnosed with tuberous sclerosis (TS). Single-photon emission computed tomography (SPECT) revealed increased blood flow in the right frontal lobe (Figure 1b). We performed magnetoencephalography (MEG) while the patient was in sedated sleep with Triclofos Sodium. We used a whole-head neuromagnetometer (NeuromagSystem, Elekta Neuromag Oy, Helsinki, Finland) that consisted of 102 identical sensors, each of which contained two planar gradiometers that were positioned at right angles to each other and one magnetometer. We analyzed the spikes using equivalent current dipole modeling and superimposed MEG spike sources (MEGSSs) on the patient’s MRI. We used local sensors to place the MEGSSs with a goodness of fit of more than 80%. MEG showed dipole sources that were distributed in the right parietotemporal areas and that were concordant with the peritubular region (Figure 1c). There was no spike source in the left hemisphere. The patient had no clinical seizures during the MEG recording. We recorded the scalp EEG with a digital sampling frequency of 500 Hz for 8 h using a Nihon Kohden Neurofax (EEG-1200) (Nihon Kohden Corporation, Tokyo, Japan) using the international 10–20 scalp electrode system. The interictal paroxysmal discharges showed repeated high-voltage polyspikes and spike and wave complexes that occurred every 10–20 s over the right centroparietotemporal areas. The adverse head motions and eye deviations to the right that were followed by spasms were recorded thrice, and the seizures involving tonic limb contractions that were not followed by spasms were recorded twice. On the ictal EEG, theta bursts preceded the adverse head motions and eye deviations. At the beginning of the periodic spasms, the recordings showed no preceding spikes but showed slow waves that were superimposed with low-amplitude fast activity over the right parietotemporal region (Figure 2a). During the periodic spasms, the EEG showed independent high-amplitude slow waves from the right and left hemispheres. We exported the scalp EEG video data sections that were recorded from around the time of the seizures with spasms and performed an MBFA using Short Spectrum Eye software (Gram Corporation, Saitama, Japan) in order to analyze the frequency and distribution of FOs. We analyzed the power spectrograms of the frequency bands between 5.7 (time constant, 0.03 s) and 120 Hz with a frequency resolution of 2 Hz and a temporal resolution of 10 ms and measured the amplitude of each frequency in order to calculate the power in μV^2^ in 26 episodes of spasms [4]. As a result, in 14 spasm episodes, the analysis showed FOs of 60–70 Hz with high spectral power that were predominantly over the right centroparietotemporal areas 100–200 ms before each spasm (Figures 2b3). In nine spasms, the records were contaminated with movement artifacts and/or muscle activity, and their interpretation was difficult. The remaining three spasms lacked clear gamma activity despite minimal artifacts and occurred mostly in the ending part of a series. This region was approximately concordant with the area around the cortical tuber that was seen on MRI, the area of high blood perfusion observed on ictal SPECT, and the cluster of spike sources observed on MEG. None of the FOs appeared at the beginning of the subsequent tonic motion (data not shown). We were not able to detect interictal high gamma activity, although we analyzed the 20 min of interictal EEG. At the age of 5 months, tuber resection was performed. Three months postoperatively, the periodic spasms had completely disappeared, and another type of brief tonic or partial myoclonus seizure that occurred once a day remained. In the analysis of the ictal EEG performed at this time, high gamma activity was not detected over the right lobes, but other high gamma activity appeared in the left central and parietal areas. One year postoperatively, the brief tonic seizures decreased to monthly, and she could sit with support. One year and seven months postoperatively, the brief tonic seizures disappeared, and she has been seizure-free.

**Figure 3:**
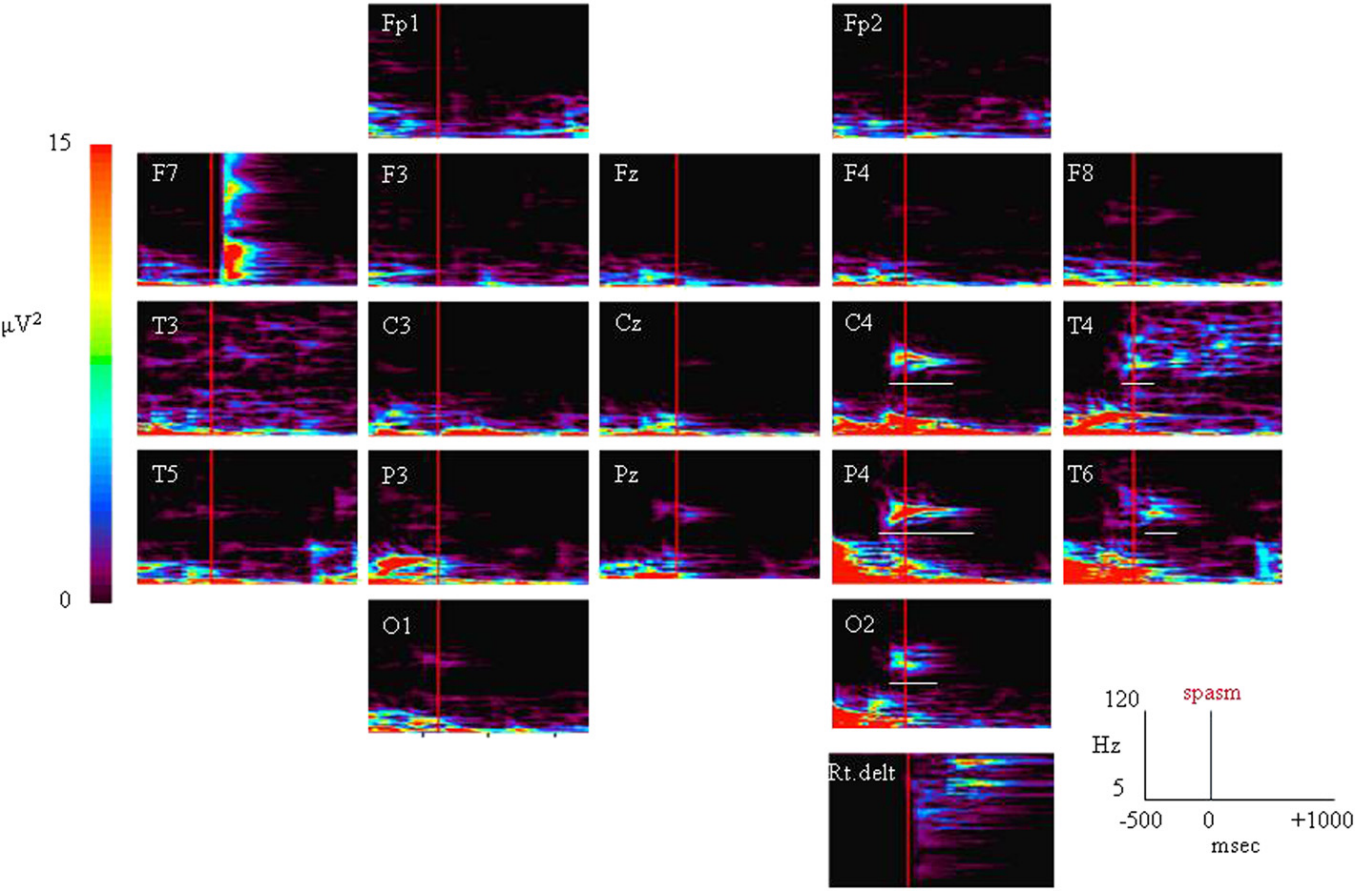
**Single frequency analysis by multiple band frequency analysis performed in the same window as in Figure**2**a.** Gamma activity of 60–70 Hz with high spectral power was seen over the right centroparietal areas 100–200 ms before spasms. This fast activity immediately spread to the right temporal area. The vertical axis indicates the frequency, the horizontal axis indicates the time course, and the colors indicate the power (μV^2^).

## Discussion

Epileptic seizures in patients with TS are often refractory to regimens of many antiepileptic drugs. Patients with TS often have multiple tubers, and it is difficult to determine the epileptogenic tuber. Moreover, not only tubers, but also nontuberous regions of cortex could be sources of the epileptogenesis because some patients with TS have diffusely decreased gray matter volumes [10,11] and diffuse cellular differentiation abnormalities [12]. Recently, MEG, SPECT, and positron emission tomography studies have pointed to specific tubers as the source of seizures [13,14], and surgical approaches involving lesionectomies of the target tubers have led to good control of the epilepsy [15,16]. Although HFO analyses are useful for detecting the source of seizures, the detection of these sources has been thought to be difficult using scalp EEG because of attenuation or dissipation by the skull and scalp, a mixture of electromyogram (EMG) artifacts, or the small region where epileptic discharges occur. Thus, intracranial EEG has typically been performed in order to detect the source of the seizures. For neonates or infants, video monitoring using intracranial electrodes is rarely performed because of the risk of accidental removal or intracranial infection. However, for young children, fast-activity analyses of scalp EEGs have been recently applied [4,7,17], and it was found that high gamma activity was associated with ictal discharge in a patient with epileptic spasms [6]. Using scalp EEG, we were able to detect and record repeated high gamma activity that had different spectra in this case compared to the EMG results that were reported previously [18]. However, because we could not detect high gamma activity of the preceding seizures, they may have involved differences in the depth of their source or in the type of epilepsies.

This report has provided evidence from scalp EEG recordings that the cortex of the surrounding tuber may be potentially epileptogenic or symptomatogenic. As previously reported, high gamma activity appears in both epileptogenic [15] and symptomatogenic regions [9], and these findings are useful for surgical resections as well as for HFOs. During the spasms, high gamma activity was generated in the rolandic area [19]. This activity appeared in the surrounding area of the right frontal tuber, and this area accorded with the location of interictal spike sources on MEG and increased blood perfusion on SPECT. These MEGSS might appear in a widespread area because they were detected during the interictal period. The electrodes that were placed above the tuber did not detect any high gamma activity, and SPECT showed decreased blood perfusion. Therefore, it was suggested that the tuber itself had no epileptic or symptomatogenic activity. However, high gamma activity was not detected around the resected tuber after the lesionectomy, and new high gamma activity arose over the contralateral lobe. In TS, after a lesionectomy, another tuber often exhibits new epileptogenicity, and thus, a lesionectomy may break the normal inhibitory system of the local epileptogenic network [15]. However, we thought we should eliminate the periodic spasms because her development had arrested after she manifested this type of seizure. Even if high gamma activity in the right hemisphere showed only symptomatogenic regions of spasms, resecting this region and disconnecting the epileptic network could contribute to a decrease in or elimination of the spasms and improve her development. The resection might change the epileptic network to generate new high gamma activity and lead to the occurrence of another type of brief tonic seizure. However, her development improved gradually and achieved the equivalence of that of a healthy six-month-old. The frequency of her seizures decreased substantially one year after the operation, and she has been seizure-free one year and seven months postoperatively.

This report provided the first evidence of an epileptogenic region in an infant with TS using MBFA on scalp EEG data. The evidence of epileptogenicity that was based on these data accorded with the MBFA of the scalp EEG was useful in detecting the epileptogenic region. Moreover, the analysis of the scalp EEG using an MBFA is useful for a precise evaluation of the epileptogenetic region and may be evidence that is useful for surgical resection. However, in our study, the grids of the scalp EEG did not have a resolution high enough to allow for an examination of the detailed seizure onset region. A device, such as a high-density EEG, is needed for a more precise analysis. In addition, we were not able to analyze the ictal delta waves because we used a low-pass filter.

## Conclusion

In conclusion, scalp EEG, which can identify the ictal onset zone by detecting gamma activity, is useful for detecting the epileptogenic region in an infant with TS.

### Consent

Written informed consent was obtained from the patient’s relatives for publication of this case report.

## Competing interests

The authors declare that they have no conflict of interest.

## Authors’ contributions

KI made a substantial contribution to the conception and design and drafted the manuscript. EN participated in the clinical management during hospitalizations and helped to draft the manuscript. RH helped to acquire and analyze the HFO data. KS and MS participated in the clinical management during the hospitalizations and revised the manuscript. TK, YK, AT, and TO are surgeons who performed the surgical interventions, and YK also acquired and analyzed the MEG data. All authors read and approved the final manuscript.
